# Indoor Smoke Exposure and Risk of Anthracosis

**Published:** 2014-11

**Authors:** Mostafa Qorbani, Masud Yunesian, Hamid Reza Baradaran

**Affiliations:** 1 Department of Public Health, Alborz University of Medical Sciences, Karaj, Iran;; 2 Department of Epidemiology, Iran University of Medical Sciences, Tehran, Iran;; 3 Department of Environmental Health, School of Public Health, Tehran University of Medical Sciences, Tehran, Iran;; 4 Center for Air Pollution Research, Institute for Environmental Research, Tehran University of Medical Sciences, Tehran, Iran;; 5 Endocrine Research Center, Institute of Endocrinology and Metabolism, Iran University of Medical Sciences, Tehran, Iran

**Keywords:** Anthracosis, Indoor air pollution, Iran

## Abstract

The association between indoor smoke exposure due to traditional baking (baking homemade bread) and anthracosis has rarely been investigated. The aim of the present study is to quantify such association among the Iranian population. A hospital based case-control study was carried out on 83 anthracotic cases and 155 controls (83 individuals with non-anthracotic pulmonary disorders from the pulmonary ward and 72 persons from the surgical ward without any known pulmonary disorders). The interview was performed using the “American Thoracic Society” questionnaire, comprising demographic information, occupational history, cigarette smoking, and indoor smoke exposure due to traditional baking. Multivariate analysis was performed by logistic regression. Comparison between cases and pulmonary ward controls showed that only the association between indoor smoke exposure due to traditional baking and anthracosis in women was statistically significant (OR: 4.30, 95% CI: 1.31 to 14.10). This was concluded after adjusting for other risk factors such as occupational exposure to dust, age, and education. When surgical ward controls were considered as control, after controlling for the significant risk factors, we found a significant relationship between indoor smoke exposure due to traditional baking and anthracosis (OR: 3.35, 95% CI: 1.49 to 7.55). Based on the findings from this study, it is concluded that there is an association between indoor smoke exposure and anthracosis. Women are significantly more susceptible to anthracosis than men are when exposed to smoke exposure.

## Introduction

Biomass fuel (wood, peat, animal dung and agricultural crop residues) is used extensively in rural areas of the developing countries such as Iran.^1^ Indoor air pollution (IAP) arising from the combustion of wood smoke and biomass fuels during baking food has been recognized as probable cause of respiratory system diseases. Some reports have also demonstrated a strong positive association between IAP and respiratory diseases.^2^^,^^3^

Anthracosis is characterized by black pigmentation of lung parenchyma and bronchial mucosa which is attributed to the inhalation of carbonaceous particles.^4^ Cigarette smoking, urban air pollution, coal mining, occupational exposure to dust and pulmonary tuberculosis have been recognized as the main factors of its etiology. According to the past case-series studies in Iran, in addition to these factors, indoor smoke exposure due to oven based traditional baking is found to be common among anthracotic cases.^5^^,^^6^ In a case-report study by Amoli, it is shown that exposure to indoor smoke due to traditional baking in ground ovens was reported extensively in the past history of anthracotic patients. In this category, women were at greater anthracosis risk due baking.^6^ Most of the past studies in Iran were case-reports without control group and unsuitable for casual association assessment.^5^^,^^6^ Consequently, this study was instigated to quantify any possible relationship between indoor smoke exposures due to ground oven based traditional baking and anthracosis.

## Patients and Methods

This case-control study was conducted between September 2009 and December 2010 in Imam Khomeini Hospital, Tehran, Iran. This hospital is the national lung referral center affiliated with Tehran University of Medical Sciences (TUMS). During this period, 83 cases were identified from Bronchoscopy unit at Imam Khomeini Hospital as bronchial anthracosis. The diagnoses of cases were based on fibrotic bronchoscopy (ICD-10 code: J60) and chest X-ray findings by an experienced pulmonologist. To prevent bias during the selection process, controls were chosen from two different sources. The first group was selected via convenience sampling method from the surgical ward at Imam Khomeini Hospital. According to their medical history and chest X-ray findings, these individuals had no known respiratory system diseases (n=72). The second group included 83 controls and was sampled via convenience sampling method among pulmonary ward patients undergone fibrotic bronchoscopy. These patients had diagnoses other than anthracosis as was reconfirmed by chest X-ray findings by an experienced pulmonologist. 

Controls were frequently matched with cases based on age (15-year interval). Verbal informed consents were provided by all patients and ethical approval was obtained by the Research Ethics Committee of TUMS.

*Data Collection*

The patients were interviewed by two trained nurses using a valid and reliable questionnaire based on the “American Thoracic Society questionnaire for epidemiologic studies”.^7^ The questionnaire comprised of demographic information, family medical history, past medical history, occupational history and cigarette smoking status. Cigarette smoking exposure was estimated in pack-years (a pack consists of 20 cigarettes). In particular, additional targeted questions were included in the questionnaire. These were on variables regarding indoor smoke exposure due to ground oven based traditional baking in terms of the onset, the duration and the frequency of exposure (per week) and other sources of smoke exposures in the homes (e.g. heating, cooking food, etc.).

*Statistical Analysis*

T-test and One-way ANOVA test were used for continuous data and Chi square for categorical data analysis between groups. The association between frequency and duration of indoor smoke exposure due to traditional baking and anthracosis was investigated using chi square for trends, in terms of duration and frequency of exposure categories. Multiple logistic regression (MLR) model using the Enter method was fitted to data to adjust for the presence of confounding and evaluation of interaction. All variables having P<0.2 in the univariate analysis were presented to the MLR model. All statistical analysis was performed in SPSS version 16 software. 

## Results

The demographic characteristics of cases and controls are shown in table 1. A total of 238 patients (83 as cases and 155 as control) were included in this study. The mean age of the patients was 54.6 years (SD: 16.4), 60% were male, approximately 11% were smokers and the mean age of smoking onset was 21.7 years (SD: 7.1).

**Table 1:** Distribution of baseline characteristics by cases and controls

**Variables **	**Cases (%)** **(n=83)**	**Pulmonary ward controls (%) ** ** (n=83)**	**Surgical ward controls (%)** **(n=72)**	**P value **
Marital status				
Single	4 (4.8)	7 (8.4)	10 (13.9)	0.10
Married	74 (89.2)	72 (86.7)	62 (86.1)	
Widowed or divorced	5 (6)	4 (4.8)	0 (0)	
Ethnicity				
Fars	43 (55.1)	35 (45.5)	26 (40.6)	0.58
Turk	23 (29.5)	25 (32.5)	26 (40.6)	
Kurd	5 (6.4)	5 (6.4)	3 (4.4)	
Others	7 (9)	12 (15.6)	9 (9)	
Sex				
Male	47 (56.6)	55 (66.3)	43 (59.7)	0.43
Female	36 (43.4)	28 (33.7)	29 (40.3)	
Cigarette smoking				
Yes	31 (37.3)	36 (43.4)	24 (33.3)	0.39
No	52 (62.7)	47 (56.6)	48 (66.7)	
Duration of cigarette smoking (year)^* ^	31.90 (15.05)	29.34(13.8)	30.43 (15.34)	0.65
Age began cigarette smoking (year)^*^	22.63 (7.62)	21.33 (6.17)	20.91 (7.85)	0.52
Age (year)^* ^	58.65 (15.78)	52.20 (16.16)	52.75 (16.60)	0.03
Education (year)^*^	4.04 (5.42)	^ ^6.03 (5.40)	5.80 (5.31)	0.01

Data for the duration of cigarette smoking, subject’s age of beginning cigarette smoking, subjects’ age and education are mean (SD).

*Association between Indoor Smoke Exposure Due to Traditional Baking and Anthracosis (Control from Surgical Ward)*

As shown in table 2, 66% of cases and 30.6% of controls from the surgical ward were exposed to indoor smoke. Indoor smoke exposure due to the traditional baking had a relatively high risk (OR: 4.46, 95% CI: 2.26 to 8.78) and the odds of occupational exposure to dust was almost three time higher (OR: 2.82, 95% CI: 1.45 to 5.49). Stratified analysis showed that, the association between indoor smoke exposure due to traditional baking and anthracosis after stratifying for potential confounding variables remained statistically significant. After deploying MLR analysis, only indoor smoke exposure due to traditional baking (OR: 3.35, 95% CI: 1.49 to 7.55) remained statistically significant variable in the model.

**Table 2:** Association between independent variables and anthracosis (control chosen from surgical ward)

**Variables **	**Cases (%) (n=83) **	**Controls (%) ** **(n=72)**	**Crude OR** **(95% CI)**	**Adjusted OR** **(95% CI)**
Indoor exposure (Y/N)^**^	55 (66.3)	22 (30.6)	4.46 (2.28-8.78)	3.35 (1.49-7.55)
Sex (F/M)	36 (43.4)	29 (40.3)	1.13 (0.60-2.15)	
Occupational exposure to dust (Y/N)	48 (58.5)	23 (33.3)	2.82 (1.45- 5.49)	1.76 (0.83-3.74)
Age (years)^*^	58.65 (15.78)	52.75 (16.60)	1.02 (1.00-1.04)	1.02 (0.99-1.03)
Cigarette smoking (pack-years)^*^	10.94 (21.14)	8.07 (18.91)	1.00 (0.99-1.02)	
Education (years)^*^	4.04 (5.42)	5.80 (5.31)	0.94(0.88-1.00)	1.02 (0.94-1.10)

^*^Data for the cigarette smoking, subjects’ age and education are Mean (SD); ^**^ Indoor smoke exposure due to baking home-made bread

Trend analysis indicated that there was an association between anthracosis and indoor smoke exposure due to traditional baking and anthracotic cases had more life span than indoor smoke exposure. Furthermore, as shown in table 3, there were more moderate (11-20 years smoke exposure) and long (>20 years smoke exposure) duration of exposure among the cases.

**Table 3:** Association between ISEBHB and anthracosis in trend analysis (control chosen from surgical ward)

**Variables**	**Cases (%) (n=83)**	**Controls (%) (n=72)**	**P value**
Indoor exposure^*^ (Y/N)	55 (66.3)	22 (30.6)	<0.001
Frequency of Indoor exposure (per week)^**^	3.71 (2.72)	2.28 (1.27)	0.04
No exposure	28 (33.7)	50 (69.4)	<0.001
Once	7 (8.4)	4 (5.6)	
Twice	20 (24.1)	12 (16.7)	
>3 times	28 (33.7)	6 (8.3)	
Duration of Indoor exposure (years)^**^	22.54 (13.7)	15.38 (13.20)	0.05
No exposure	28 (33.7)	50 (69.4)	<0.001
0-10 years	14 (16.9)	15 (20.8)	
11-20 years	20 (24.1)	3 (4.2)	
20+ years	21 (25.3)	4 (5.6)	

^**^Indoor smoke exposure due to baking home-made bread; ^**^Data for the frequency of indoor smoke and duration of indoor smoke are mean (SD)

*Association between Indoor Smoke Exposure Due to Traditional Baking and Anthracosis (Control from Pulmonary Ward)*

The association between anthracosis and indoor smoke exposure due to traditional baking were stratified by gender and were significantly different; for men (OR: 1.02, 95% CI: 0.45 to 2.32), for women (OR: 6.00, 95% CI: 1.99 to 18.04) and the homogeneity test suggested significant interaction (P<0.01). The crude OR_S _for females (univariate analysis) is shown in table 4. The estimated crude ORs for indoor smoke exposure due to traditional baking were (OR: 6.00, 95% CI: 1.99 to 18.04), occupational exposure to dust (OR: 3.00, 95% CI: 0.84 to 10.63), age (OR: 1.03, 95% CI: 1.00 to 1.07) and education (OR: 0.90, 95% CI: 0.81 to 1.00), respectively. Multiple logistic regression analysis showed that only indoor smoke exposure due to traditional baking (OR: 4.30, 95% CI: 1.31 to 14.10) remained as statistically significant variable in the model. The crude OR_S _for males are shown in table 5. Multiple logistic regression analysis was not performed, since the results of univariate analyses showed that none of the independent variables (even indoor smoke exposure) have P<0.2.

**Table 4:** Association between independent variables and anthracosis in female (control chosen from pulmonary ward)

**Variables **	**Cases (%) ** **(n=36) **	**Controls (%) ** **(n=36)**	**Crude OR** **(95% CI)**	**Adjusted OR** **(95% CI)**
Indoor exposure (Y/N)^**^	24 (66.7)	7 (25)	6.00 (1.99- 18.04)	4.30 (1.31-14.10)
Occupational exposure to dust (Y/N)	12 (33.3)	4 (14.3)	3.00 (0.84-10.63)	1.35(0.27- 6-66)
Age (years)^*^	58.58 (15.15)	50.42 (16.42)	1.03 (1.00-1.07)	1.01 (0.97-1.05)
Cigarette smoking (pack-years)^*^	1.89 (11.33)	3.07 (13.17)	0.99 (0.95-1.03)	
Education (years)^*^	3.14 (5.06)	5.77 (5.13)	0.90 (0.81-1.00)	0.95 (0.84-1.07)

^*^Data for the cigarette smoking, subjects’ age and education are mean (SD); ^**^Indoor smoke exposure due to baking home-made bread

**Table 5:** Association between independent variables and anthracosis in male (control chosen from pulmonary ward)

**Variables **	**Cases (%) ** **(n=47) **	**Controls (%) ** **(n=55)**	**Crude OR** **(95% CI)**
Indoor exposure (Y/N)^**^	31 (66)	36 (65.5)	1.02 (0.45-2.32)
Occupational exposure to dust (Y/N)	36 (76.6)	38 (69.1)	1.46 (0.60-3.54)
Age (years)^*^	58.70 (16.41)	53.61 (15.99)	1.02 (0.99-1.04)
Cigarette smoking (pack-years)^*^	21.62 (28.92)	25.21 (32.70)	0.99 (0.98-1.00)
Education (years)^*^	4.72 (5.64)	6.16 (5.56)	0.95 (0.88-1.02)

^*^Data for the cigarette smoking, subjects’ age and education are mean (SD); ^**^ Indoor smoke exposure due to baking homemade bread

## Discussion

The findings from the present study demonstrate a positive association between indoor smoke exposure due to traditional baking and anthracosis (particularly in women) being consistent with the past studies.^8^^,^^9^ A case-report study by Amoli, reported on non-smoker anthracotic cases with prolonged exposure to smoke while baking homemade bread using ground ovens in their homes.^7^ A report by Aslani et al. showed that among 96 anthracotic cases, 30% with a past history of indoor smoke exposure due to traditional baking,^5^ while Torun et al. reported all patients were exposed to biomass fuel.^8^

It is also found that, among the population considered in the present study, indoor smoke exposure due to traditional baking is the main risk factor for anthracosis. This finding is consistent with Dennis et al. results that demonstrated nearly 50% of obstructive airway disease (OAD) cases might be attributable to long-term wood smoke exposure.^10^

In the present study, the result of indoor smoke exposure effect on anthracosis between control groups (surgical and pulmonary) was different. The presence of some respiratory diseases (e.g. tuberculosis) in the pulmonary control group may explain this discrepancy. Since pulmonary tuberculosis is recognized as a risk factor for anthracosis, it may justify such discrepancy.^9^

There is a gap between IAP dose-response pattern and respiratory diseases. In a recent meta-analysis, Kurmi et al. stated that among 23 reviewed studies, none provided information regarding the trend pattern.^11^ In the present study, the association between indoor smoke exposure due to traditional baking and anthracosis in trend analysis was evident. The mean duration of traditional baking (in years) and weekly time spent (in days) was significantly higher among anthracotic cases. These findings are consistent with Dennis et al. study regarding the association between OAD and wood smoke being dominant in trend analysis.^10^

The effect of modification by gender, which was seen between indoor smoke exposure due to traditional baking and anthracosis (with a stronger association among females than males) may originate from systematic bias in the assessment of exposure-response relationship. Ezzati and Kammen mentioned that adjusting for the amount of cooking activity, eliminates the statistical association of gender. This confirms that the role of gender was a proxy of exposure pattern.^12^ In other word, since traditional baking is of the sole responsibility of women in the rural areas of Iran, probably the reported cases of direct exposure by women is more intense than men. Thus, the length and intensity of exposure is crucial for exposure assessment model in IAP. According to their definition, the term “length” refers to the amount of household cooking or baking activities that a person performs. The term “intensity of exposure” refers to the concentration of smoke that a person inhale while in the proximity of an oven or stove. Another explanation for women’s greater response to indoor smoke exposure may originate from their bronchial hyper-responsiveness to air pollution.^13^ This was in-line with the observation made in the past studies on air pollution and respiratory diseases.^14^^,^^15^

The present study contains certain limitations. Primary limitation is related to the absence of exposure measurement device for unbiased study of participants. Similar to other case-control studies, information gathering was based on patient’s recollection, which is prone to error. Since this study was a hospital-based case-control study, therefore selection bias could be considered in such type of study. Since comparison groups were selected among different patients with dissimilar diseases from the same hospital, potential bias in our result is reduced to an absolute minimum. 

It is known that various types of fuels such as wood, animal dung and agricultural residues are used for traditional baking among the Iranian families in the rural areas. These are typically used simultaneously or in random sequence. Inability to determine the exact type of fuel used for traditional baking was an important limitation of the present study. Consequently, indoor smoke exposure to traditional baking was considered as the main independent variable. 

In the present study, we were unable to control the effect of the genetic factor. Furthermore, confidence interval was wide in some instances and thus, it is recommended that further study should include a larger number of patients and controls. 

## Conclusion

This study provides confirmation that indoor smoke exposure due to traditional baking is related to the risk of anthracosis. Iranian women are more susceptible than men are to anthracosis when exposed to smoke exposure. 
